# Validity and reliability of the Persian version of the machiavellian personality scale and its association with social adjustment and risky behaviors in Iranian college students

**DOI:** 10.1186/s12888-023-05177-x

**Published:** 2023-09-25

**Authors:** Mitra Asadi, Somayeh Yoosefi

**Affiliations:** 1 Department of Clinical Psychology, Sanandaj Branch, Islamic Azad University, Sanandaj, Iran; 2 Department of Education, Faculty of Education and Psychology, Azarbaijan Shahid Madani University, Tabriz, Iran

**Keywords:** Personality, Psychometrics, Social adjustment, Risky behaviors

## Abstract

**Objective:**

The present study aims to develop and assess the psychometric properties of the Persian version of the Machiavellian Personality Scale (P-MPS), and evaluate its relationship with social adjustment and risky behaviors in Iranian college students.

**Methods:**

Participants were 500 healthy college students (270 females and 230 males) from medical and non-medical universities in Sanandaj, Iran. They completed the P-MPS, the social adjustment subscale of the Bell Adjustment Inventory, and the Youth Risk Behavior Surveillance System (YRBSS) questionnaire. The factor structure of the P-MPS was evaluated by exploratory factor analysis followed by confirmatory factor analysis (CFA). Cronbach’s alpha coefficient was used to examine the internal consistency of the P-MPS and Pearson correlation test was used to investigate the relationship of the P-MPS score with the scores of social adjustment subscale and YRBSS.

**Results:**

The P-MPS showed good content validity (Content validity ratio = 0.73, content validity index = 0.90), construct validity, and internal consistency (α = 0.802). The CFA results supported the four-factor solution of the questionnaire. The total score of P-MPS and its dimensions showed a significant negative relationship with social adjustment (p < 0.05). Moreover, its score was significantly correlated with risky behaviors (p < 0.05).

**Conclusion:**

The Persian version of MPS can be used for assessing Machiavellianism in the Iranian population.

## Introduction

Young people engage more in risky behaviors (e.g. alcohol/drug abuse, unsafe sex, bullying, and suicide) when facing problems which can threaten their health [1]. The factors that cause risk behaviors in youth include biological factors (e.g., family history of drug addiction, higher intelligence), socioeconomic factors, personality (e.g., low self-esteem), and behaviors such as engagement in school activities [2]. Negative personality traits can lead to an increase in risk-taking activities [3–8]. One of these negative personalities is Machiavellian personality. Machiavellianism is one of the traits in what is called the Dark Triad. This personality trait is characterized by “manipulation and exploitation of others, an absence of morality, unemotional callousness, and a higher level of self-interest” [9, 10]. People with high level of Machiavellianism are less agreeable and conscientious [11, 12]. They create alliances, and for maintaining a good reputation they do everything they can. They do not fully break the rules, but have a remarkable ability to evade them cleverly [13, 14]. Therefore, they are often called “chameleons” and “wolves in sheep’s clothing” ]15]. They prefer not to form close romantic relationships due to the lack of emotional attachment; sexual and working relationships provide opportunities for them to emotionally manipulate others; e.g., by coercion, inducting pleasure, and subsequent reward [15].

Machiavellianism is associated with different problematic behaviors and acts as a risk factor for later social adjustment. “In the technical language of psychology, getting along with the members of society as best one can is called social adjustment” [16]. It is also defined as “the degree to which an individual engages in competent social behavior and adapts to the immediate social context” [17]. According to American Psychological Association dictionary, social adjustment is “accommodation to the demands, restrictions, and mores of society, including the ability to live and work with others harmoniously and to engage in satisfying interactions and relationships”. It is an attempt made by a person to address the norms and values of a society to be accepted [18]. The DSM-5 defined the basic characteristics of a person with anti-social personality disorder as negligence and infringement on the rights of others, expressed in irresponsibility, absence of self-accusation, lack of compassion and aggressiveness [19, 20]. These traits are interrelated with the aspects of Dark Triad including Machiavellianism.

Timely assessment of Machiavellian behaviors in young people is important for avoiding risky behaviors and social maladjustment in them. The trait of Machiavellianism alone is commonly measured by the Mach-IV scale developed by Christie and Geis in 1970 [21]. It is a self-report scale which assesses three distinct themes: “the use of deceit in interpersonal relationships, a cynical view of human nature, and the lack of morality” [22]. Based on its score, those with high Machiavellian trait are called “High Machs” and those with low Machiavellian trait are labeled as “Low Machs’’. Dahling et al. [10] identified some drawbacks in the Mach-IV such as inconsistent reliability, ambiguity in factor structure, and the use of many poor questions (e.g., double-barreled questions). In this regard, they developed a new tool for assessment of Machiavellianism named the Machiavellian Personality Scale (MPS). The MPS has 16 items assessing amoral manipulation, desire for control, desire for status, and distrust of others. Amoral manipulation is “a willingness to disregard standards of morality and see value in behaviors that benefit the self at the expense of others”; desire for control is “a need to exercise dominance over interpersonal situations to minimize the extent to which others have power”; desire for status is “a desire to accumulate external indicators of success”; and distrust of others is “a cynical outlook on the motivations and intentions of others with a concern for the negative implications that those intentions have for the self” [10]. Machiavellianism can also be measured using the related subscale in dark triad assessment tools including Short Dark Triad [13] and Dirty Dozen [23].

It is important to examine the psychometric properties of personality assessment tools in different cultural contexts, particularly for cross-cultural comparisons. There are Korean and Portuguese versions of MPS [24, 25], but we found no validated Persian version of MPS. Due to the lack of an instrument to solely assess Machiavellian behaviors in Iranian population, and the lack of study on the relationship of Machiavellianism with social adjustment in Iranian youth, this study aims to develop and examine the psychometric properties of the Persian version of MPS, and assess the association of Machiavellianism with social adjustment and risky behaviors in Iranian youth.

## Materials and Methods

### Participants

In this study, participants were 500 healthy college students (with no physical disability and mental illness and not using psychiatric drugs according to self-report) from medical and non-medical universities in Sanandaj (A Kurdish city in Iran) who were selected using a convenience sampling method in spring 2017. They included 270 females and 230 males. Most of them had age 24–30 years (n = 234, 46.8%) and were undergraduate students (n = 307, 61.4%). Inclusion criteria were being a college student in Sanandaj city, no any physical disability or mental disease, not using psychiatric drugs according to self-report, and willingness to participate in the study. The exclusion criteria were the return of an incomplete questionnaire and lack of cooperation.

### Tools

The MPS has 16 items assessing Amorality (items 1–5), Desire for Control (items 6–8), Desire for Status (items 9–11), and Distrust of Others (items 12–16). The items are rated on a 5-point Likert scale from 1 = strongly disagree 5 = to strongly agree. The score for amorality ranges 5–25; for desire for control, 3–15; for desire for status, 3–15; and for distrust of others, 5–25. The total score ranges from 16 to 80, where higher scores indicate higher Machiavellianism. According to Dahling et al. [10], MPS is a valid and reliable tool for assessing Machiavellianism. They reported its good reliability (α = 0.82).

The Bell Adjustment Inventory (BAI), developed by Bell in 1962 [26], has 140 items designed to measure home adjustment, health adjustment, social adjustment, and emotional adjustment. They are answered by “Yes” or “No”. A high score indicates a great number of bad symptoms in a given area. In this study, we used the social adjustment subscale of BAI which has 32 items in its Persian version answered by “Yes”, “No”, or “No idea” according to Michaeli Manee and Madadi Emamzadeh [27]. Its total score ranges from 0 to 64. A high score indicates a desire to withdraw from the community and a low score indicates a tendency to aggression in social relations. The social adjustment subscale has a reliability of α = 0.88 [28]. In our study, the Cronbach’s alpha for the social adjustment subscale was obtained 0.79 which is acceptable.

The Youth Risk Behavior Surveillance System (YRBSS) questionnaire designed by the Centers for Disease Control and Prevention, is used to monitor priority health risk behaviors that contribute to the leading causes of mortality, morbidity, and social problems among youths and adults. We used the Persian version of YRBSS which was validated by Baheiraei et al. [29]. It has 94 items, three items surveying race (Persian, Kurdish, Turkish, Arab, Lor), height and weight without shoes, five items about safe driving, 11 about violence-related behaviors, two items about bullying, five items about sad feelings and attempted suicide, four items about cigarette smoking, three items about electronic vapor products such as e-cigarettes and e-cigars, e-hookahs, three items about other tobacco products, four items about drinking alcohol, two items about number of alcoholic drinks in a row, three items about marijuana use, 11 about other drugs, nine items about sexual behavior, two items about body weight, 12 about foods or drinks during the past seven days, six items about physical activity, one item about concussions (a blow or jolt to the head), and eight items about other health-related topics. Baheiraei et al. [29] reported that, in overall, 97.75% of the YRBSS items had moderate to excellent reliability to be used in Iranian population.

### Procedure

This study consisted of three main steps: Translation, administration, and psychometric analysis. The “forward-backward” technique was used to translate the MPS from English into Persian. A psychologist and PhD student in English language translated the questionnaire into Persian and then back translated into English by a health psychologist and a professional translator. The final version was developed after a consensus by 10 psychologists and psychiatrists. After explaining the study objectives to the participants and obtaining informed consent from them, the final Persian version of MPS (P-MPS) was administered to them. They also completed the social adjustment subscale of BAI as well as the YRBSS questionnaire.

To assess the content validity of the P-MPS, it was sent to 10 faculty members and they were asked to rate each item on a scale as “essential,” “useful but not essential,” or “not necessary”. Then, the Content validity Ratio (CVR) was calculated based on Lawshe (1975)’s method [30]. Furthermore, experts were asked to rate instrument items on a 4-point scale in terms of relevancy to the study construct (1 = not relevant, 2 = somewhat relevant, 3 = quite relevant, and 4 = highly relevant). For calculating the content validity index (CVI) of items, the number of experts giving a rating of “highly relevant” for each item was divided by the total number of experts. Its values range from 0 to 1, where I-CVI > 0.79 indicates that the item is relevant; 0.70–0.79, the item needs revisions, and if < 0.70, the item is eliminated [31]. The Average Scale-level CVI (S-CVI/Ave) was calculated by taking the sum of the I-CVIs divided by the total number of items [32]. A S-CVI/Ave ≥ 0.9 show excellent content validity [33].

The factor structure of the P-MPS was evaluated by exploratory factor analysis (EFA; varimax rotation). To confirm the factor structure described by EFA, the confirmatory factor analysis (CFA) was conducted in AMOS software on different data set obtained from 200 participants. Cronbach’s alpha coefficient was used to examine the internal consistency of the P-MPS. Pearson correlation test was used to investigate the relationship of the P-MPS score with the scores of social adjustment subscale of BAI and YRBSS.

## Results

### Descriptive statistics

The normal distribution of the of the study variables was first examined using the standardized skewness and kurtosis values. Acceptable values of skewness fall between − 3 and + 3, and kurtosis is appropriate from a range of − 10 to + 10 when utilizing SEM [34]. Table 1 presents the mean scores of the P-MPS, social adjustment, YRBSS, and their skewness and kurtosis values. In our study, all three variables were at acceptable ranges for factor analysis. The mean score of social adjustment subscale was 11.05 ± 5.26 (ranging from 1 to 24) indicating their low to moderate social adjustment level, and the mean overall score of YRBSS was 68.28 ± 24.34 (ranging from 26 to 208).

**Table 1:** Mean and standard deviation of the P-MPS scores

	Component	Mean	Standard Deviation	Min	Max	skewness	kurtosis
Machiavellianism	Amoral manipulation	14.58	2.82	8	24	-0.016	-0.222
	Desire for control	11.26	2.17	6	15		
	Desire for status	10.53	1.95	6	15		
	Distrust of others	16.25	2.70	10	24		
	Total P-MPS	52.63	6.75	32	70		
Social adjustment	BAI	11.05	5.26	1	24	0.537	-0.269
Risky behaviors	YRBSS	68.28	24.34	26	208	1.757	5.744

### Content validity

The CVR values for the overall instrument and its subscales of amoral manipulation, desire for control, desire for status, and distrust of others were obtained 0.73, 0.80, 0.75, 0.69, and 0.70, respectively. Since the number of panelist was 10, the CVR > 0.62 indicates acceptable level. The CVR values were higher than 0.62; hence, it can be said that the P-MPS had good content validity. Table 2 presents the CVR for items and I-CVI of relevance. As can be seen, all I-CVI values were > 0.79, indicating that all items were relevant. The S-CVI/Ave was obtained 0.908, indicating excellent content validity.

**Table 2:** The values of CVR for items and I-CVI of relevance

Item	CVR	I-CVI
1	0.62	0.94
2	0.75	0.96
3	0.70	0.90
4	0.65	0.87
5	0.70	0.95
6	0.69	0.90
7	0.75	0.87
8	0.70	0.94
9	0.65	0.89
10	0.69	0.85
11	0.73	0.90
12	0.70	0.93
13	0.78	0.90
14	0.75	0.96
15	0.69	0.88
16	0.65	0.89

### Factorial validity

For assessing the facture structure of the P-MPS, first we conducted Kaiser-Meyer-Olkin (KMO) test and Bartlett’s test of sphericity. A KMO value over 0.5 and a significance level for the Bartlett’s test below 0.05 suggest a substantial correlation. The results reported a KMO value of 0.815 and Bartlett’s test results were as following: X^2^ = 1785.988, df = 120, and p < 0.001. The KMO value was between 0.8 and 1 indicating that the sampling is adequate for EFA. The significance level for the Bartlett’s test was less than 0.05; hence, we can reject the null hypothesis and say that there is a correlation between data and the items of P-MPS are suitable for EFA. Using principal components analysis, four factors were extracted, which are shown in Table 3. As can be seen, the first factor explained 14.92% of variance; the second factor explained 14.84% of variance; the third factor explained 12.46% of variance; and the fourth factor explained 11.80% of variance. These factors were amoral manipulation, desire for control, desire for status, and distrust of others, respectively. They together explained 54.03% of the variance. The results of varimax rotation are presented in Table 4. As can be seen, five items loaded on the first factor, three items on the second factor, three items on the third factor, and six items on the fourth factor. Tabachnick and Fidell [35] suggested factor loading cut-offs of 0.32 (poor), 0.45 (fair), 0.55 (good), 0.63 (very good) or 0.71 (excellent). Based on these criteria, nine items of the P-MPS had excellent factor loading; four items had very good factor loading; two items had good factor loading, and one item (no. 12) had fair factor loading.

**Table 3:** Results of principal components analysis

Component	Initial Eigenvalues			Extraction Sums of Squared Loadings			Rotation Sums of Squared Loadings		
	Total	% of Variance	Cumulative %	Total	% of Variance	Cumulative %	Total	% of Variance	Cumulative %
1	4.084	25.527	25.527	4.084	25.527	25.527	2.388	14.925	14.925
2	1.768	11.049	36.576	1.768	11.049	36.576	2.375	14.843	29.768
3	1.436	8.974	45.550	1.436	8.974	45.550	1.994	12.461	42.229
4	1.357	8.484	54.033	1.357	8.484	54.033	1.889	11.805	54.033

**Table 4:** Rotated component matrix

Item	Factor			
	1	2	3	4
1	0.630			
2	0.777			
3	0.571			
4	0.793			
5	0.528			
6		0.759		
7		0.751		
8		0.768		
9			0.764	
10			0.712	
11			0.832	
12				0.472
13				0.694
14				0.660
15				0.716
16				0.675

**Fig. 1:**
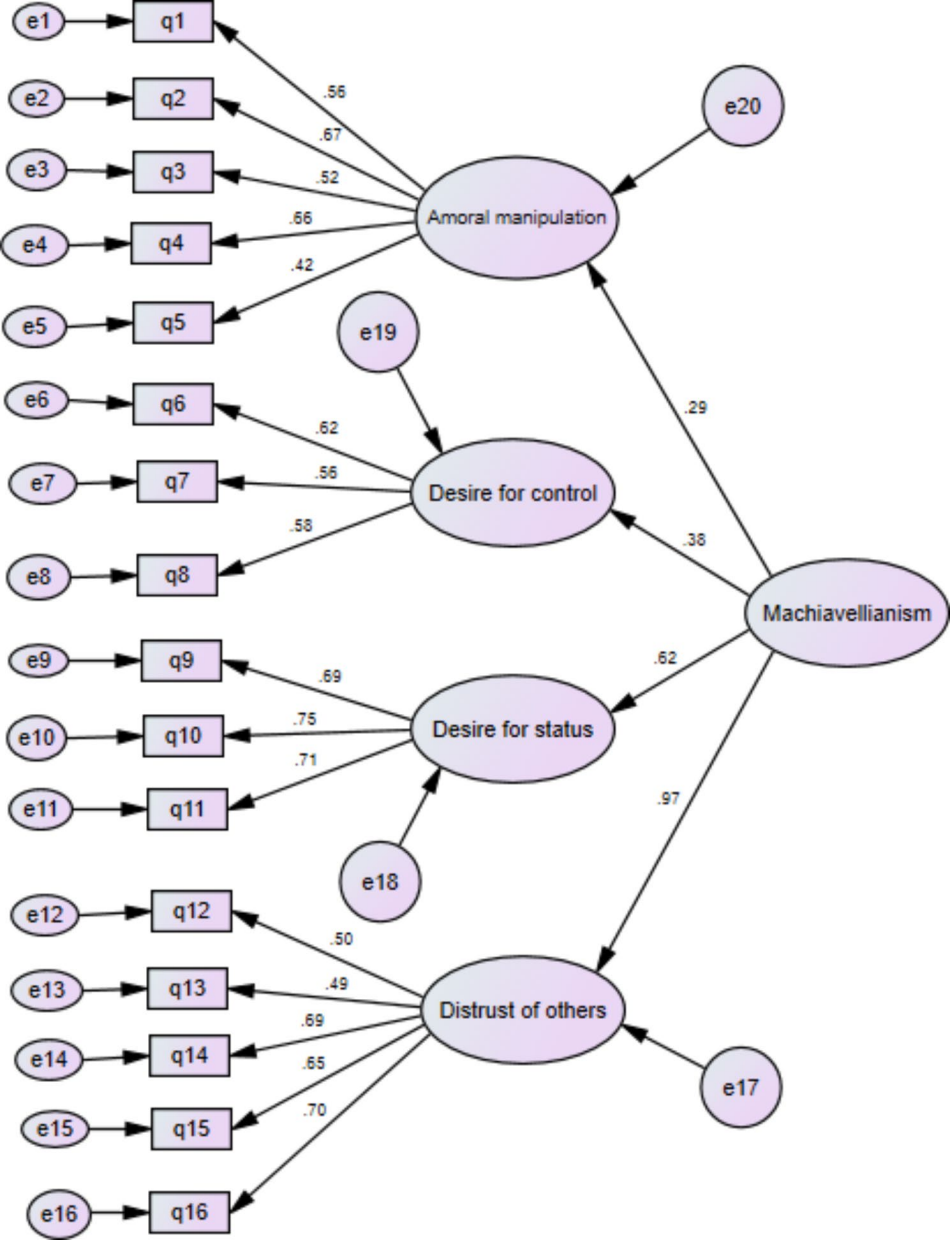
CFA model of the P-MPS and its path coefficients

The CFA results confirmed the four-factor structure of P-MPS described by EFA. The 4-factor model showed good fit across all fit indices: P = 0.025, X^2^/df = 1.296, comparative fit index (CFI) = 0.95, normed fit index (NFI) = 0.83, non-normed fit index (NNFI) = 0.94, incremental fit index (IFI) = 0.95, adjusted goodness of fit index (AGFI) = 0.90, and root-mean-square error of approximation (RSMEA) = 0.039. According to the literature, good or acceptable fit thresholds for these indices are RMSEA < 0.08, CFI ≥ 0.90, AGFI ≥ 0.90, and NNFI ≥ 0.90. The CFA mode of 4-factor solution is illustrated in Fig. 1, and the specifications of the model are presented in Table 5.

**Table 5:** Estimates of parameters in the SEM model

Path	Estimate	Std. Error	Critical ratio	Sig.
Machiavellianism -> Amoral manipulation	1			
Machiavellianism -> Desire for control	1.168	0.522	2.235	0.025
Machiavellianism > Desire for status	2.995	1.135	2.639	0.008
Machiavellianism -> Distrust of others	2.782	1.242	2.240	0.025

### Internal consistency

Internal consistency of the overall P-MPS was very good (α = 0.802). For the subscales of amoral manipulation (α = 0.70), and distrust of others (α = 0.70), desire for status (α = 0.73), and desire for control (α = 0.70), it was good and acceptable.

### Association with social adjustment

Pearson correlation test results for assessing the association between the scores of P-MPS and social adjustment subscale of BAI (Table 6) showed a significant negative correlation between total score of P-MPS and social adjustment (R=-0.582, p < 0.01). The P-MPS dimensions of distrust of others (R=-0.350, p < 0.01), amoral manipulation (R=-0.249, p < 0.01), desire for control (R=-0.375, p < 0.01), and desire for status (R=-0.751, p < 0.01) also showed a significant negative relationship with social adjustment.

**Table 6:** Pearson correlation coefficients for the association between the P-MPS score and social adjustment

	Amoral manipulation	Desire for control	Desire for status	Distrust of others	Machiavellianism	Social adjustment
**Amoral manipulation**	1					
**Desire for control**	0.248*	1				
**Desire for status**	0.267*	0.257*	1			
**Distrust of others**	0.376*	0.373*	0.323*	1		
**Machiavellianism**	0.726*	0.651*	0.612*	0.772*	1	
**Social adjustment**	-0.249*	-0.375*	-0.751*	-0.350*	-0.582*	1

* p < 0.001

### Association with risky behaviors

Pearson correlation test results for assessing the association between the scores of P-MPS and YRBSS (Table 7) showed a positive significant correlation between their overall scores (R = 0.93, p = 0.000). Moreover, all YRBSS domains had a positive significant correlation with the total score of P-MPS (p < 0.001).

**Table 7:** Pearson correlation test results for the association between the scores of P-MPS and YRBSS

Independent variable	Dependent variable	R	Sig.
Machiavellianism	Safe driving	0.46	0.000
	Violence-related behaviors	0.46	0.000
	Bullying	0.57	0.000
	Sad feelings and attempted suicide	0.15	0.000
	Cigarette smoking	0.46	0.000
	Vapor products	0.31	0.000
	Other tobacco products	0.44	0.000
	Drinking alcohol	0.54	0.000
	Number of alcoholic drinks	0.47	0.000
	Marijuana use	0.27	0.000
	Other drugs	0.34	0.000
	Sexual behavior	0.55	0.000
	Body weight	0.12	0.000
	Foods or drinks	0.45	0.000
	Physical activity	0.43	0.000
	Concussions	0.20	0.000
	Other health-related topics	0.20	0.000
	Total	0.93	0.000

## Discussion

This study was conducted to assess the psychometric properties of the Persian version of MPS, one of the novel instruments to measure Machiavellianism. The results showed that the Persian version of MPS had good content validity (CVR = 0.73), factorial structure or construct validity, and internal consistency (α = 0.802) to measure Machiavellianism in Iranian samples. Based on CFA results, the 4-factor model had good fit across all fit indices. Our results are consistent with results for the English, Korean, and Portuguese versions of MPS [10, 24, 25] which had four-factor structures. Dahling et al. [10] reported a Cronbach’s alpha of 0.82 for the main (English) version tested on 167 university students. Kim et al. [24] reported an alpha value of 0.79 for the Korean version tested on 339 university students, and Grohmann and Battistella [25] reported a minimum alpha value of 0.60 for the Portuguese version tested on 264 employees of an organization.

Results revealed the significant negative association of the total score of P-MPS and its dimensions with social adjustment. Based on the results, it can be said that those with a desire for amoral manipulation, control of others, receiving respect and deference from others, and with no trust on others are more likely to socially maladjusted. We found no study that have investigated the correlation between Machiavellianism and social adjustment to compare the results. Kedzuch and Williams [36] in a study showed that Machiavellianism was associated with destructive coping strategies such as behavioral disengagement. Maladaptive coping strategies can lead to social maladjustment.

The results of the present study also showed the significant association of P-MPS score with risky behaviors (YRBSS score) in college students. Quednow et al. [37] also showed that Machiavellianism is higher in cocaine users, whereas Jauk and Dieterich [6] in a review study concluded that Machiavellianism is not associated with substance-related and non-substance-related addictive behaviors. In the studies by Jones and Neria [3] and Van Geel et al. [5], Machiavellianism positively predicted hostility and traditional bullying which is consistent with our results.

There were some limitations in this study including non-assessment of criterion validity, convergent/divergent validity, test-retest reliability, and interrater reliability of the P-MPS due to the time constraints. More studies are recommended to assess these properties of the P-MPS. This study was conducted in one city of Iran (Sanandaj, as a Kurdish city); hence, the generalization of findings regarding the personality profile, social adjustment, and risky behaviors of participants to all college students in Iran should be done with caution. The use of P-MPS is recommended in interventional studies on other age groups or for assessing the difference in Machiavellian personalities of men and women in Iran.

## Conclusion

The Persian version of MPS is a valid and reliable tool, and can be used for assessing Machiavellianism in Iranian college students. Machiavellianism among college students in Iran is associated with their social adjustment and risky behaviors.

## Data Availability

The datasets used and analyzed during the current study are not publicly available (due to individual privacy), but are available from the corresponding author on reasonable request.
